# Dynamical determinants enabling two different types of flight in cheetah gallop to enhance speed through spine movement

**DOI:** 10.1038/s41598-021-88879-0

**Published:** 2021-05-05

**Authors:** Tomoya Kamimura, Shinya Aoi, Yasuo Higurashi, Naomi Wada, Kazuo Tsuchiya, Fumitoshi Matsuno

**Affiliations:** 1 Department of Electrical and Mechanical Engineering, Nagoya Institute of Technology, Nagoya, Japan; 2 Department of Aeronautics and Astronautics, Graduate School of Engineering, Kyoto University, Kyoto, Japan; 3 Laboratory of System Physiology, Joint Faculty of Veterinary Medicine, Yamaguchi University, Yamaguchi, Japan; 4 Department of Mechanical Engineering and Science, Graduate School of Engineering, Kyoto University, Kyoto, Japan

**Keywords:** Computational biology and bioinformatics, Biological physics

## Abstract

Cheetahs use a galloping gait in their fastest speed range. It has been reported that cheetahs achieve high-speed galloping by performing two types of flight through spine movement (gathered and extended). However, the dynamic factors that enable cheetahs to incorporate two types of flight while galloping remain unclear. To elucidate this issue from a dynamical viewpoint, we developed a simple analytical model. We derived possible periodic solutions with two different flight types (like cheetah galloping), and others with only one flight type (unlike cheetah galloping). The periodic solutions provided two criteria to determine the flight type, related to the position and magnitude of ground reaction forces entering the body. The periodic solutions and criteria were verified using measured cheetah data, and provided a dynamical mechanism by which galloping with two flight types enhances speed. These findings extend current understanding of the dynamical mechanisms underlying high-speed locomotion in cheetahs.

## Introduction

**Figure 1:**
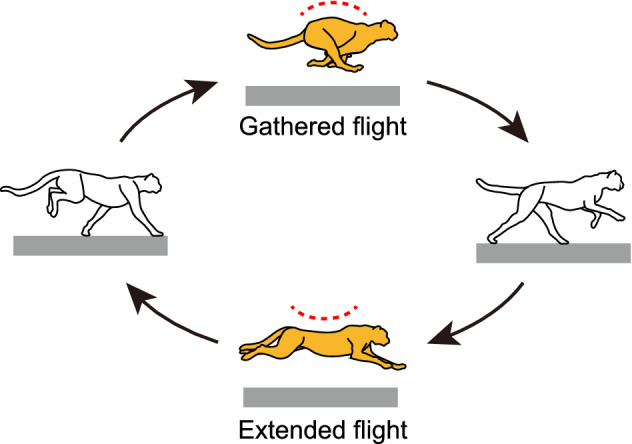
Cheetah galloping involves two types of flights through spine movement: gathered and extended.

Cheetahs are the fastest land animal. They use a galloping gait in their highest speed range, that involves two types of flight in each gait cycle: gathered and extended^1–3^ (Fig. 1). Gathered flight occurs after the liftoff of the forelimbs, in which the forelimbs and hindlimbs are underneath the body, towards the midline. Extended flight occurs after the liftoff of the hindlimbs, in which the forelimbs and hindlimbs are extended outwards. These limb movements increase stride length, enhancing gait speed. While cheetahs exhibit two flight types, many animals, including horses, exhibit only one type of flight (generally gathered flight), and their gait speed is slower than that of cheetahs. Extended flight allows cheetahs to use the acceleration produced via the ground reaction forces of the hindlimbs effectively to achieve high-speed locomotion^1^. Furthermore, cheetahs exhibit remarkable spine movement during the flights, which further enhances gait speed. Specifically, the spine movement differs between the two types of flight; whereas the spine is flexed during the gathered flight, it is extended during the extended flight (Fig. 1), which swings the limbs further and increases the stride length^4–8^. Although cheetahs are known to achieve high-speed locomotion using two types of flight and spine movements, the dynamic factors that enables the two types of flight and spine movement while galloping remain unclear.

Animal running consists of the flight phase and stance phase, which are governed by different dynamics. During the flight phase, all feet are in the air and the center of mass (COM) of the whole body exhibits ballistic motion. Conversely, during the stance phase, some of the feet are in contact with the ground and the body receives ground reaction forces. Because of the complex and hybrid nature of the governing dynamics, there are limitations to the understanding of the dynamical mechanisms underlying animal running that can be gained solely from observations of animals. To overcome the limitations of the observational approach, modeling approaches have recently attracted research attention^1,9–15^. Because the legs can be represented by springs, a monopode vertical hopping model was proposed^16^. A spring-loaded inverted pendulum (SLIP) model was then developed to investigate the mechanisms of animal gait from a dynamic perspective, particularly for human running^17–22^. The SLIP model has been improved for examining gait in quadrupeds^23–28^ to clarify the common and unique principles between the animal gaits. Recently, the SLIP model has been further improved to investigate the dynamic roles of spine movement in quadruped running^29–35^. However, few researchers have focused on the types of flight and spine movement during galloping, and the dynamical conditions under which the cheetah gallop involves two different flight types through spine movement remain unclear.

To clarify these dynamical conditions, we developed a simple analytical model, which focused on the vertical movement and the spine movement based on our previous study^32,33^. We obtained possible periodic solutions with two different flight types through spine movement (like cheetah galloping), and solutions with only one flight type (unlike cheetah galloping). The periodic solutions provided two criteria determining the flight type, which are related to the position and magnitude of ground reaction forces entering the body. The periodic solutions and criteria were verified with the help of measured data from cheetahs, and provided the dynamical mechanism by which galloping involving two flight types produces higher-speed locomotion compared with galloping involving one flight type. We discuss the biological relevance of our findings.

## Results

### Model

**Figure 2:**
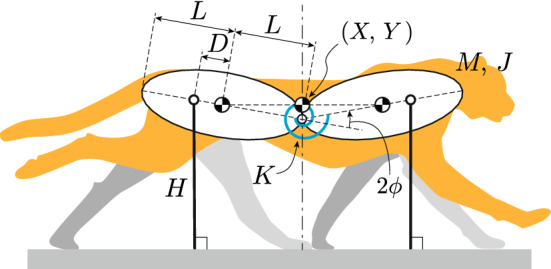
Model comprising two rigid bodies and two massless bars. The bodies are connected by a joint with a torsional spring. The bars represent the legs.

We developed a two-dimensional mathematical model emulating the main dynamical characteristics of a cheetah consisting of two rigid bodies and two massless bars (Fig. 2). The bodies are connected by a joint, which is modeled to emulate the bending movement of the spine and has a torsional spring with a spring constant of *K*. The bars represent the legs. We assumed that the fore and hind parts of the model have the same physical parameters. *X* and *Y* are the horizontal and vertical positions, respectively, of the COM of the whole body. The spine joint angle is represented by $2\phi$. The mass and moment of inertia around the COM of each body are *M* and *J*, respectively. The lengths of each body and leg bar are 2*L* and *H*, respectively. The distance between the COM and the root of the leg bar, which is the joint between the leg bar and the body, is *D*. *D* is positive when the root is outside the COM relative to the spine joint. The root positions were not determined by the root joints of animal legs, but by the net ground reaction forces, because the leg bars of the model represent the effect of net ground reaction forces as impulsive forces, rather than the animal legs themselves. Therefore, *D* indicates the impulse position. The torsional spring is at its equilibrium position when the fore and hind bodies are in a straight line ($\phi =0$). The gravitational acceleration is *g*.

**Figure 3:**
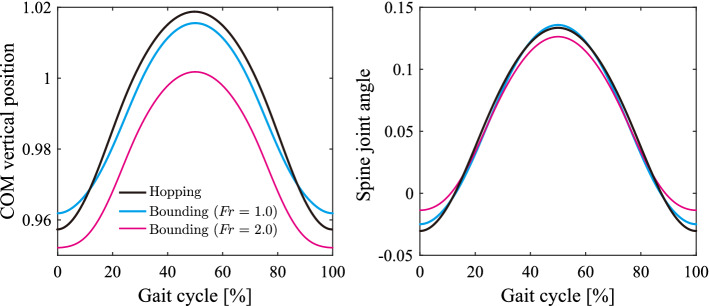
Comparison of COM vertical and spine movements between hopping and bounding in our previous model, modified from Kamimura et al.^33^ $Fr = (\mathrm{averaged\ velocity})/\sqrt{gL_0}$ indicates Froude number, where $L_0$ is the leg length of the model.

Although cheetah galloping invoves a large spine movement, the pitching movement of the line connecting the root of the neck and the hip, that is the pitching movement of the whole body, is relatively small. In our previous work^32^, we used a model comprising of two rigid bodies and two legs, which was able to perform the spine and whole body pitching movements, as well as horizontal and vertical movements. The simulation results revealed that the vertical movement of the COM and spine movement were significant determinants of the dynamic characteristics of bounding gait, compared with the whole body pitching and horizontal movements. Specifically, when we compared the COM vertical and spine movements between hopping (without the whole body pitching and horizontal movements) and bounding (with the whole body pitching and horizontal movements) using our previous model, significant differences were not observed (Fig. 3). Furthermore, even when we ignored the horizontal ground reaction force in our previous work^33^, the principal dynamic characteristics in bounding gait remained unchanged. In the current study, we neglected the horizontal ground reaction force in the model and assumed that the leg bars were always perpendicular to the ground, which allowed us to ignore the dynamics of *X*. In addition to the horizontal movement, we also neglected the pitching movement of the whole body, making the COM vertical positions of the fore and hind bodies identical, and the fore and hind legs touch the ground simultaneously. That is, our model focuses on the vertical hopping movement. Although this movement differs from actual galloping in some ways, the COM vertical and spine movements are not significantly affected by this assumption, because the dynamical effects of the foot contacts by the fore and hind legs on these movements are identical, as explained in more depth in Supplemental Information S1.

### Possible periodic solutions

Because cheetah galloping involves two flight phases and two stance phases per gait cycle, we obtained analytical periodic solutions with two flight phases and two foot contacts for each gait cycle based on the linearized equations of the governing equations of the model. We defined the periodic solution as ${\hat{q}}(\tau ) = [{\hat{y}}(\tau ) \ {\hat{\phi }}(\tau )\ \dot{{\hat{y}}}(\tau ) \ \dot{{\hat{\phi }}}(\tau )]^\top$ ($0 \le \tau < \tau _1+\tau _2$), where $y=Y/H$, $\tau = t/\sqrt{H/g}$, $j=J/(MH^2)$, $k=K/(MgH)$, $\omega = \sqrt{2k/j}$, and $d = D/H$; $\tau =0$ is the onset time of the first flight phase, $\tau _1$ and $\tau _2$ are the durations of the first and second flight phases, respectively, and $\dot{*}$ indicates the derivative of variable $*$ with respect to $\tau$. We assumed that $\tau _i<2\pi /\omega$ ($i=1,2$) because animals do not oscillate their spines more than once in one gait cycle. The periodic solution was derived by

1a $$\begin{aligned} {\hat{y}}(\tau )&= {\left\{ \begin{array}{ll} -\frac{1}{2}\tau ^2 + a_1 \tau + b_1, &{} 0 \le \tau< \tau _1 \\ -\frac{1}{2}(\tau -\tau _1)^2 + a_2 (\tau -\tau _1) + b_2, &{} \tau _1 \le \tau < \tau _1+\tau _2 \end{array}\right. } \end{aligned}$$

1b $$\begin{aligned} {\hat{\phi }}(\tau )&= {\left\{ \begin{array}{ll} c_1 \cos (\omega \tau + \psi _1), &{} 0 \le \tau< \tau _1 \\ c_2 \cos (\omega (\tau -\tau _1) + \psi _2), &{} \tau _1 \le \tau < \tau _1+\tau _2 \end{array}\right. } \end{aligned}$$

where $a_i$, $b_i$, $c_i>0$, $-\pi \le \psi _i < \pi$, and $\tau _i$ ($i=1,2$) are given as functions of the initial phase $\psi _1$ and the amplitude $c_1$ of the spine movement in the first flight phase, as shown in Supplemental Information S2. $\psi _1$ and $c_1$ satisfy $\Gamma (\psi _1,c_1)=0$, where

2 $$\begin{aligned} \Gamma (\psi _1, c_1) = {\left\{ \begin{array}{ll} -\dfrac{\psi _2(\psi _1, c_1)}{\omega }-a_2(\psi _1, c_1), &{} -\pi \le \psi _2(\psi _1, c_1)< 0 \\ \dfrac{\pi -\psi _2(\psi _1, c_1)}{\omega }-a_2(\psi _1, c_1). &{} 0 \le \psi _2(\psi _1, c_1) < \pi \end{array}\right. } \end{aligned}$$

Therefore, the periodic solution is determined by one free parameter ($\psi _1$ or $c_1$) and three physical parameters (*j*, *d*, *k*). These parameters are determined using measured data from cheetahs, as described below.

### Classification of solutions

The flight phases are classified into two types based on the spine joint movement: gathered and extended. In gathered flight, the spine joint is flexed (${\hat{\phi }}<0$) at the mid-flight phase. Therefore, $\dot{{\hat{\phi }}}<0$ is satisfied at the beginning of gathered flight. In extended flight, the spine joint is extended (${\hat{\phi }}>0$) at the mid-flight phase. Therefore, $\dot{{\hat{\phi }}}>0$ is satisfied at the beginning of extended flight. Because periodic solutions have two flight phases per gait cycle, periodic solutions are classified into four types (both gathered, both extended, first gathered and second extended, and first extended and second gathered). In addition, some periodic solutions have two identical flights, which satisfy ${\hat{q}}(\tau )={\hat{q}}(\tau +\tau _1)$, $a_1 = a_2$, $b_1=b_2$, $c_1=c_2$, $\psi _1=\psi _2$, and $\tau _1 = \tau _2$. We distinguished such solutions from those that had two different flights, and classified them into two types (two identical gathered and two identical extended). The solutions of these two types are identical to those obtained in our previous work^33^.

**Figure 4:**
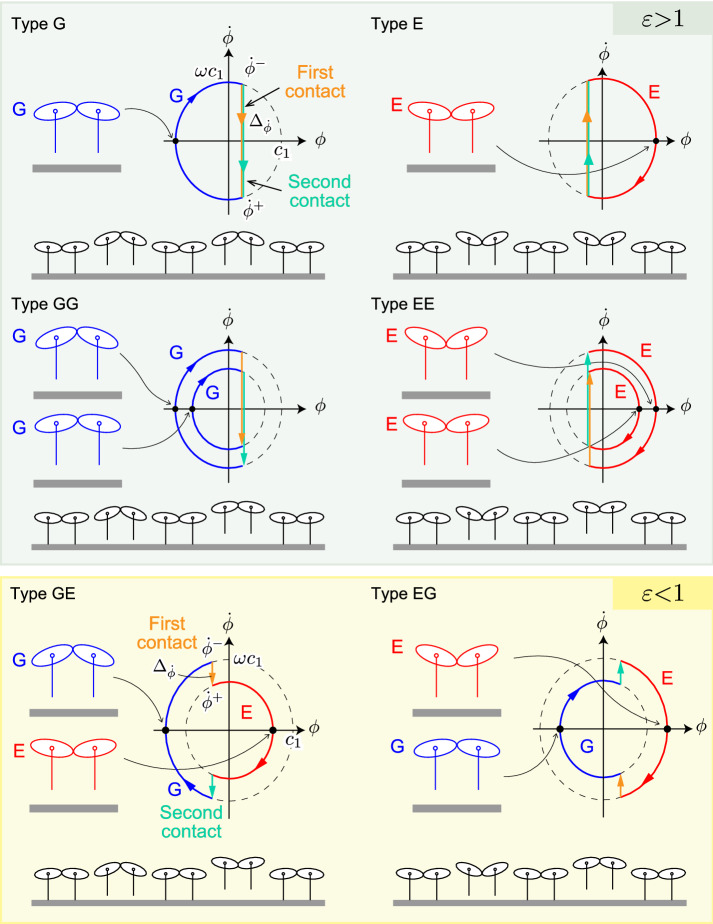
Phase plane of spine joint angle $\phi$ and snapshots of six types of solutions. G and E indicate gathered and extended flights, respectively. $\Delta _{{\dot{\phi }}}$ is the difference between ${\dot{\phi }}^+$ and ${\dot{\phi }}^-$, which indicate angular velocities immediately prior to and following foot contacts, respectively. $\omega c_1$ is the amplitude of angular velocity. $\varepsilon$ is ratio of the angular velocity change ($\varepsilon = \Delta _{{\dot{\phi }}}/\omega c_1$). When $\varepsilon >1$, solutions have one type of flight (solutions of type G, E, GG, and EE). Solutions of type GE and EG satisfy $\varepsilon <1$.

As a result, the periodic solutions are classified into the following six types, as shown in Fig. 4:

1. Type G: Two identical gathered flights
2. Type E: Two identical extended flights
3. Type GG: Two different gathered flights
4. Type EE: Two different extended flights
5. Type GE: Two different flights (first: gathered, second: extended)
6. Type EG: Two different flights (first: extended, second: gathered).

We distinguished types GE and EG with the assumption that the amplitude of oscillation of the spine joint angle $\phi$ in the first flight phase is greater than that of the second flight phase ($c_1>c_2$).

The periodic solution provides an important criterion to determine the solution type; the signs of the spine movement angular velocities $\dot{{\hat{\phi }}}^-$ and $\dot{{\hat{\phi }}}^+$ are different for one type of flight (solutions of types G, E, GG, and EE) but are identical for two different types of flights (solutions of types GE and EG), where $*^-$ and $*^+$ indicate the state immediately prior to and following foot contacts, respectively. This means that, while the direction of the spine movement changes with each foot contact for one type of flight, the direction does not change for two types of flights.

### Evaluation of solutions using measured cheetah data

**Figure 5:**
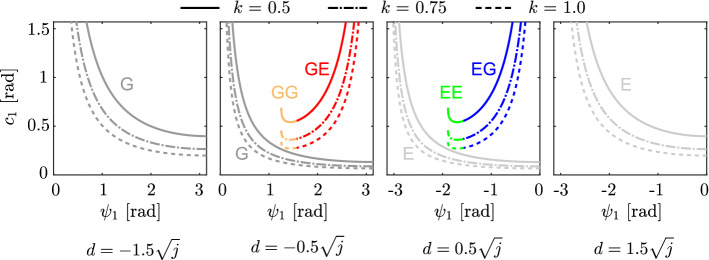
Relationships between $\psi _1$ and $c_1$ for periodic solutions with various values of *k* and *d* in the model. The solution type depends little on *k*, but substantially depends on *d*.

From the measured cheetah data, we determined $M=19$ kg, $J=0.53$ $\hbox {kgm}^2$, and $H=0.67$ m (*M* is comparable to the measured data in Hudson et al.^36^), which resulted in $j=0.063$. Figure 5 shows the relationship between the initial phase $\psi _1$ and the amplitude $c_1$ that satisfies $\Gamma (\psi _1, c_1)=0$ and produces the solution for various values of the impulse position *d* and the torsional spring constant *k*, by using the value of the moment of inertia *j* obtained from the measured data. Specifically, we used $d=-1.5\sqrt{j}$, $-0.5\sqrt{j}$, $0.5\sqrt{j}$, and $1.5\sqrt{j}$, and $k=0.5$, 0.75, and 1.0. The solution type depended little on *k*, largely depending on *d*. For $d=-1.5\sqrt{j}$ and $1.5\sqrt{j}$, only type G and only type E exist, respectively, and the solution is unique for $\psi _1$ and $c_1$. Conversely, for $d=-0.5\sqrt{j}$, types GG and GE exist, as well as type G, and for $d= 0.5\sqrt{j}$, types EE and EG exist, as well as type E. For these values of *d*, the solution is not necessarily unique for $\psi _1$ or $c_1$.

**Figure 6:**
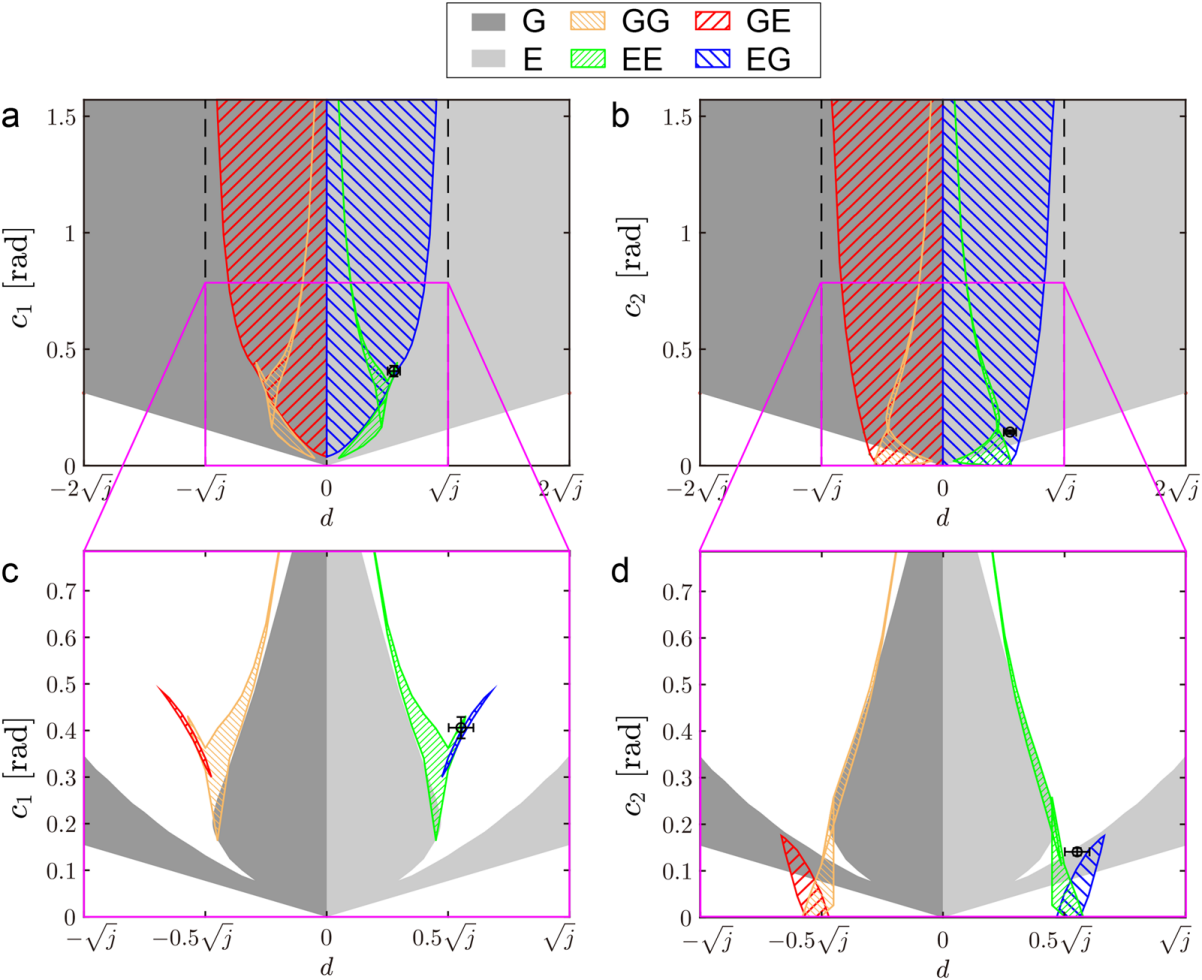
Solution type for *d* and $c_1$ (**a**), and *d* and $c_2$ (**b**). Stable solutions among solutions in a focused range of *d* and $c_1$ (**c**), and *d* and $c_2$ (**d**). Black circles and error bars show average values and standard errors of measured animal data, respectively.

To more clearly show the dependence of the solution type on the impulse position *d*, Fig. 6a shows the solution types obtained for *d* and $c_1$ by projecting the solutions in the *d*-$c_1$-$\psi _1$ space to the *d*-$c_1$ plane, where we used the torsional spring constant $k=0.80$ ($K=98$ Nm/rad) based on Hudson et al.^36^. Figure 6b shows solutions for *d* and $c_2$ in a similar way to Fig. 6a. Because the spine is never bent at a right angle during galloping, we showed the range $0\le c_i\le \pi /2$ ($i = 1,2$). Solutions with two identical flights appear when $d \ne 0$. Specifically, types G and E exist for $d<0$ and $d>0$, respectively. Conversely, solutions with two different flights appear when $-\sqrt{j}<d<\sqrt{j}$, including $d=0$. Specifically, types GG and GE exist for $-\sqrt{j}<d<0$, types GE and EG exist for $d=0$, and types EE and EG exist for $0<d<\sqrt{j}$. Detailed explanations of the dependence of the solutions on these parameters are provided in Supplemental Information S3. These results indicate that the impulse position *d* is another criterion to determine the solution type.

To further clarify important characteristics of the solutions from a dynamic viewpoint, Fig. 6c,d show stable solutions from a focused range among the solutions in Fig. 6a,b, respectively. These stable solutions were compared with the measured cheetah data (black circles). While type EG had a small region for stable solution, the measured cheetah data were located close to the stable region, as shown in Fig. 6c,d. The measured data were also located close to the stable region of type EE.

### Evaluation of two criteria

We obtained two criteria to determine the solution type. Specifically, the first criterion showed that the impulse position *d* has to satisfy $-\sqrt{j}< d < \sqrt{j}$ to achieve two different flight types. The other criterion showed that the signs of the spine movement angular velocities $\dot{{\hat{\phi }}}^-$ and $\dot{{\hat{\phi }}}^+$ have to be identical at each foot contact. We evaluated these criteria using the measured cheetah data.

First, we obtained $d=0.17\pm 0.02$ (S.E.) and $\sqrt{j} = 0.31$, which satisfied $-\sqrt{j}< d < \sqrt{j}$. The second criterion was evaluated using the ratio $\varepsilon = \Delta _{{\dot{\phi }}}/\omega c_1$, where $\Delta _{{\dot{\phi }}}$ and $\omega c_1$ indicate the angular velocity change and the amplitude of the angular velocity, respectively (Fig. 4). When $\varepsilon >1$, solutions have one type of flight. Conversely, solutions with two different flight types have $\varepsilon < 1$. We obtained $\varepsilon = 0.61 \pm 0.08$ ($\Delta _{{\dot{\phi }}} = 1.13 \pm 0.16$, $\omega c_1 = 1.84 \pm 0.11$) from the measured cheetah data. The periodic solution showed $\varepsilon = 0.49$ ($\Delta _{{\dot{\phi }}} = 0.90$, $\omega c_1 = 1.8$) by using the average of measured data for the impulse position *d* and the spine movement amplitude $c_1$. Both the measured data and periodic solutions satisfied $\varepsilon < 1$.

### Evaluation of flight duration

**Figure 7:**
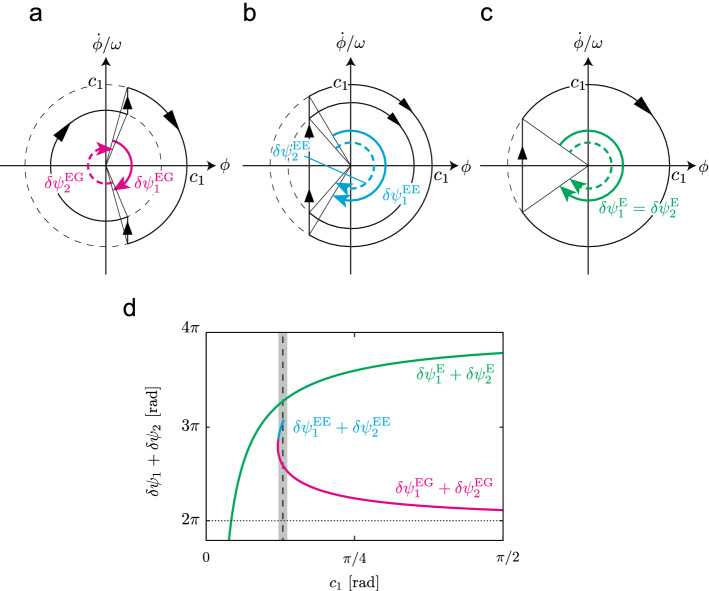
Changes $\delta \psi _i$ ($i=1,2$) of phase angle of spine joint angle $\phi$, for solutions of (**a**) type EG, (**b**) type EE, and (**c**) type E. (**d**) $\delta \psi _1+\delta \psi _2$ for amplitude $c_1$ of spine joint angle $\phi$ for periodic solutions. Dashed line is average measured $c_1$ values in cheetahs, and gray area shows standard error.

Short gait cycle durations allow animals to kick the ground frequently for acceleration and achieve high-speed locomotion^36^. From this viewpoint, we investigated the flight phase duration of the periodic solutions, which corresponds to the gait cycle duration in our model. The flight phase duration is given by $(\delta \psi _1+\delta \psi _2)/\omega$, where $\delta \psi _1$ and $\delta \psi _2$ are changes of the phase angle of the spine joint angle during the first and second flight, respectively, and are determined by $\psi _1$ and $\psi _2$, respectively. Specifically, for solutions of type EG, $\delta \psi _1^{\mathrm{EG}} = |2\psi _1^{\mathrm{EG}}|$ and $\delta \psi _2^{\mathrm{EG}}=|2\pi -2\psi _2^{\mathrm{EG}}|$, as shown in Fig. 7a, where $*^i$ indicates the constant in the solution of type *i*. For solutions of type EE, $\delta \psi _1^{\mathrm{EE}} = |2\psi _1^{\mathrm{EE}}|$ and $\delta \psi _2^{\mathrm{EE}} = |2\psi _2^{\mathrm{EE}}|$, as shown in Fig. 7b. For solutions of type E, $\delta \psi _1^{\mathrm{E}} = \delta \psi _2^{\mathrm{E}} = |2\psi _1^{\mathrm{E}}|$, as shown in Fig. 7c. Figure 7d shows $\delta \psi _1+\delta \psi _2$ for the amplitude $c_1$ of the spine joint angle $\phi$. While $\delta \psi _1+\delta \psi _2$ is close to $2\pi$ for the solution of type EG, it is much larger than $2\pi$ for the solutions of types E and EE in the range of measured values of the spine movement amplitude $c_1$ obtained from cheetahs. When $c_1$ is identical between solutions of types EG, EE, and E, solutions of type EG have the shortest flight duration among these solution types.

## Discussion

In the current study, to clarify the dynamical conditions under which cheetah gallop can involve two different flight types through the spine movement in one gait cycle, we developed a simple analytical model and derived periodic solutions. The results revealed six types (G, E, GG, EE, GE, and EG) of possible periodic solutions (Fig. 4). These solutions were classified into two types according to their flights: types G, E, GG, and EE involved one type of flight, and types GE and EG involved two different flight types.

### Conditions under which cheetah gallop involves two different types of flight

We investigated cheetah galloping, focusing on flight and spine movement. Specifically, the cheetah gallop involves two different types of flight (gathered and extended); the spine is flexed in gathered flight and extended in extended flight. Periodic solutions of types EG and GE involved two different types of flight through spine movement, similar to cheetah galloping. Conversely, periodic solutions of types E, G, EE, and GG involved only one type of flight. We verified the obtained solutions from the comparison with measured data from cheetahs. In particular, the impulse position *d* and the spine movement amplitudes $c_1$ and $c_2$ of the measured data of cheetahs were close to those of the stable solutions of the type EG (Fig. 6b,c). This result suggests that the solutions of the type EG correspond to cheetah galloping. However, the solutions of the types E and EE also exist for the measured *d* (Fig. 6), implying that cheetah gallop does not necessarily involve two types of flight. Our results suggest that cheetahs select the gallop with two types of flight because it can achieve faster locomotion than galloping with only one type of flight, as described later.

The periodic solution led to two important criteria to determine the solution type, which are related to the position and magnitude of the ground reaction force entering the body. First, the periodic solution revealed that the types of solution depend on the relationship between the impulse position *d* and the value $\sqrt{j}$, which is called the radius of gyration^37^. (The radius of gyration is the distance from the COM to the point at which the whole mass of the rigid body could be concentrated without changing its moment of inertia.) Specifically, solutions with two different flight types appear when $|d|<\sqrt{j}$ (Fig. 6), meaning that they are obtained when the impulse position is located inside the radius of gyration (the proofs are shown in Supplemental Information S3). We evaluated this criterion using the measured cheetah data and showed that cheetah galloping satisfies this condition (Fig. 6). Second, the periodic solution showed that whereas the signs of the spine angular velocities ${\dot{\phi }}^-$ and ${\dot{\phi }}^+$ are identical for two different types of flights, they are different for one type of flight (Fig. 4). This criterion suggests that the effect of the ground reaction force is too small to change the direction of the spine movement in cheetah galloping. To evaluate this criterion, we calculated the angular velocity change $\Delta _{{\dot{\phi }}}$ of the spine movement caused by the ground reaction force and the amplitude $\omega c_1$ of the angular velocity, and obtained the ratio $\varepsilon = \Delta _{{\dot{\phi }}}/\omega c_1$ in the derived solutions and measured cheetah data. When the direction of the spine movement does not change, $\varepsilon <1$ because $\Delta _{{\dot{\phi }}}$ is smaller than $\omega c_1$. Conversely, when $\varepsilon >1$, the direction changes because $\Delta _{{\dot{\phi }}}$ is larger than $\omega c_1$ (Fig. 4). We achieved $\varepsilon < 1$ for both the solutions and measured data. This result suggests that cheetahs exhibit large spine movement to reduce the effect of the ground reaction force, which prevents the direction from changing. This allows cheetah galloping to generate two different flight types.

It has been reported that the relationship between the impulse position *d* and the radius of gyration $\sqrt{j}$ is crucial in quadrupedal walking and running. When $0<d<\sqrt{j}$, the energy efficiency in walking and running (ambling) is improved^38^. This is because the moment of inertia is relatively small, and the body is easy to rotate. In this study, the obtained periodic solutions showed that when $|d|<\sqrt{j}$, the model can achieve types EG and GE, which involve large spine movements.

Furthermore, the periodic solutions enabled us to discuss the galloping gait of other animals involving only one type of flight. For example, horse galloping involves only gathered flight and rarely or never involve extended flight^1,5^. Previous observational studies have suggested that horses do not exhibit substantial bending of the spine^1,5^. Periodic solutions suggest that horses exhibit only one type of flight because the spine movement is small and the direction of the spine movement changes by the ground reaction force during the stance phase, in contrast to cheetah galloping.

### Different flight types enable high-speed locomotion

In cheetah galloping, whereas ground reaction forces caused by the hindlimbs act to accelerate, those caused by the forelimbs act to decelerate. Because extended flight occurs after the hindlimb stance, the top-speed is achieved when the hindlimbs leave the ground and the speed is maintained until the forelimbs touch the ground. Without the extended flight, the top-speed cannot be achieved because the forelimbs touch the ground before the hindlimbs leave the ground^1^. Therefore, the use of the extended flight in addition to the gathered flight achieves a higher speed of locomotion. The dynamical roles of fore and hind legs are identical in our model because we used an anterior-posterior symmetric model and ignored the whole body pitching movement. That is, our model focused only on vertical hopping. However, there was not any significant difference in dynamical properties, such as spine movement, between hopping (without whole body pitching and horizontal movements) and bounding (with whole body pitching and horizontal movements) in our previous model^33^ (Fig. 3). This suggests that the dynamical essence of cheetah galloping is explained by hopping. The current findings extend current understanding of how cheetahs achieve high-speed locomotion.

Furthermore, short gait cycle durations allow animals to kick the ground frequently for acceleration and achieve high-speed locomotion^36^. Comparison between the periodic solutions and measured cheetah data showed that the solutions of types E and EE, which have only one flight type, would be available for cheetahs (Fig. 6). However, the periodic solutions revealed that including two different flight types induced shorter gait cycle durations compared with including only one flight type (Fig. 7). Our results suggest that cheetah galloping uses two different flight types to achieve higher-speed locomotion.

## Conclusion

The current study revealed that our simple model has six types of solutions (E, G, EE, GG, EG, and GE) depending on the spine movement during the flight phase and that the impulse position and the change of the spine angular velocity determine the type of solution. In particular, stable solutions of type EG had similar characteristics to the measured data from cheetahs and reproduced cheetah galloping from the perspective of spine movement. The obtained solutions suggested the dynamical mechanism by which the galloping with two flight types enhances speed. However, because our model neglected the dynamics in horizontal movement, our model is limited in terms of understanding gait speed. Furthermore, although it has also been suggested that cheetahs achieve high-speed locomotion by extended flight so that the touchdown of the forelimbs does not decelerate in the horizontal direction^1^, our model neglects whole body pitching movement, which induced simultaneous touchdown between the fore and hind legs. In addition, our model involved several assumptions that differed from real cheetah galloping, including massless legs, instantaneous stance phases, and anterior-posterior symmetry of the model. We plan to incorporate these factors to improve our model in future studies.

Furthermore, although we focused on cheetah galloping with two different flight types, many animals, including horses, exhibit galloping with one type of flight. The mechanism underlying this difference remains unclear. In future, we plan to investigate common and specific mechanisms underlying different types of galloping gait by improving our model using measured animal data.

Our model incorporated a torsional spring connecting two rigid bodies. Previous animal data suggest that animals use their bodies as elastic structures, such as the tendons in the torso^9,39^. However, trunk muscles are also effectively used as actuators to produce energy for acceleration^2,5^. Moreover, spine movement improves energy efficiency because energy is stored in the elastic elements of the body, then released^9,39,40^. Finally, in future studies, we also intend to investigate the effect of trunk control on locomotion speed and energy efficiency.

## Methods

### Governing equations of the model

During the flight phase, the equations of motion for the COM vertical position *Y* and the spine joint angle $\phi$ are given by

3a $$\begin{aligned} 2M{\ddot{Y}}&= -2Mg, \end{aligned}$$

3b $$\begin{aligned} (2J+2ML^2\sin ^2\phi ) \ddot{\phi }&= -4K\phi - ML^2{\dot{\phi }}^2\sin 2\phi . \end{aligned}$$

When the foot touches the ground, it receives the ground reaction force. Because the COM vertical positions are identical between the fore and hind bodies, the foot contact of the fore and hind legs occurs simultaneously. This condition is given by

4 $$\begin{aligned} R(Q^-)= Y^- + D \sin \phi ^- - H = 0, \end{aligned}$$

where $Q=[Y\ \phi \ {\dot{Y}}\ {\dot{\phi }}]^\top$. Because the duty factor in animal galloping is small^36^, we assumed that the stance phase is sufficiently short and that the foot contact can be regarded as an elastic collision, involving energy conservation and no position change. The relationship between the states immediately prior to and immediately following the foot contact is given by

5a $$\begin{aligned} {\dot{Y}}^+&= -\frac{J-MD^2\cos ^2\phi ^-}{J+MD^2\cos ^2\phi ^-}{\dot{Y}}^- -\frac{2JD\cos \phi ^-}{J+MD^2\cos ^2\phi ^-}{\dot{\phi }}^-, \end{aligned}$$

5b $$\begin{aligned} {\dot{\phi }}^+&= -\frac{2MD\cos ^2\phi ^-}{J+MD^2\cos ^2\phi ^-}{\dot{Y}}^- +\frac{J-MD^2\cos ^2\phi ^-}{J+MD^2\cos ^2\phi ^-}{\dot{\phi }}^-. \end{aligned}$$

The derivation of these equations is presented in Supplemental Information S4.

In this study, we solved these governing equations under the condition $|\phi |\ll 1$ and $|{\dot{\phi }}|\ll 1$ using non-dimensional variables and parameters. The linearization of the equations of motion (3) gives

6a $$\begin{aligned} \ddot{y}&= -1 \end{aligned}$$

6b $$\begin{aligned} \ddot{\phi }&= - \omega ^2\phi . \end{aligned}$$

where $\dot{*}$ indicates the derivative of variable $*$ with respect to $\tau$, and $\ddot{*}$ indicates the second derivative of variable $*$ with respect to $\tau$. The foot-contact condition (4) is approximated by

7 $$\begin{aligned} r(q^-)= y^- + d \phi ^- - 1 = 0, \end{aligned}$$

where $q=[y\ \phi \ {\dot{y}}\ {\dot{\phi }}]^\top$. The foot-contact relationship (5) is linearized by

8 $$\begin{aligned} q^+ = Bq^-, \end{aligned}$$

where

$$\begin{aligned} {B} = \begin{bmatrix} 1 &{} 0 &{} 0 &{} 0 \\ 0 &{} 1 &{} 0 &{} 0 \\ 0 &{} 0 &{} -\dfrac{j-d^2}{j+d^2} &{} -\dfrac{2jd}{j+d^2} \\ 0 &{} 0 &{} -\dfrac{2d}{j+d^2} &{} \dfrac{j-d^2}{j+d^2} \end{bmatrix}. \end{aligned}$$

### Derivation of periodic solution

We defined the periodic solution as ${\hat{q}}(\tau ) = [{\hat{y}}(\tau ) \ {\hat{\phi }}(\tau )\ \dot{{\hat{y}}}(\tau ) \ \dot{{\hat{\phi }}}(\tau )]^\top$ ($0 \le \tau < \tau _1+\tau _2$). Because the foot-contact condition is satisfied at the first foot contact ($\tau =\tau _1$) and second foot contact ($\tau =\tau _1+\tau _2$), (7) gives

9 $$\begin{aligned} r({\hat{q}}^-(\tau _1))&=0, \end{aligned}$$

10 $$\begin{aligned} r({\hat{q}}^-(\tau _1+\tau _2))&=0. \end{aligned}$$

From the foot-contact relationship (8) and periodic condition, we obtain

11 $$\begin{aligned} {\hat{q}}^+(\tau _1)&= B {\hat{q}}^-(\tau _1), \end{aligned}$$

12 $$\begin{aligned} {\hat{q}}(0)&= B{\hat{q}}^-(\tau _1+\tau _2). \end{aligned}$$

**Figure 8:**
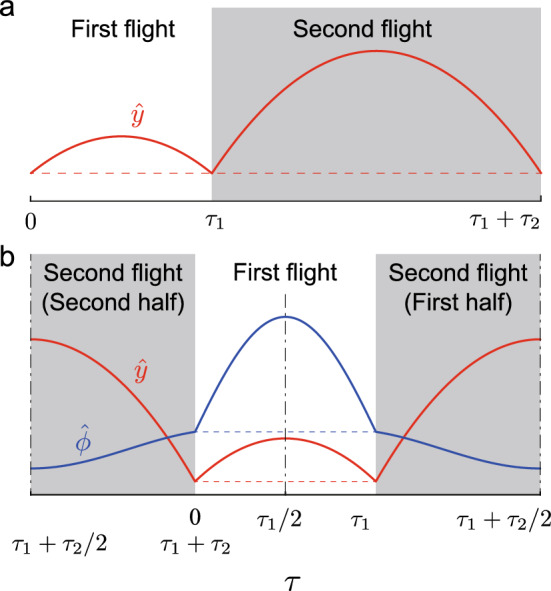
Symmetry condition for periodic solution. (**a**) ${\hat{y}}^+(\tau _1) = {\hat{y}}(0)$ for ${\hat{y}}(\tau )$. (**b**) Symmetric periodic solutions with respect to $\tau =\tau _1/2$ and $\tau = \tau _1 + \tau _2/2$.

From the conditions (9)–(12), we determined ten constants $a_i$, $b_i$, $c_i$, $\psi _i$, and $\tau _i$ ($i=1,2$) to obtain the periodic solution. However, these conditions produced various types of solutions, including solutions that are unlikely in animals. Therefore, we focused on solutions which satisfy

13 $$\begin{aligned} {\hat{y}}^+(\tau _1)={\hat{y}}(0), \end{aligned}$$

so that the COM vertical position remained unchanged at each foot contact, as shown in Fig. 8a. Under this assumption, the periodic solution is symmetric with respect to $\tau =\tau _1/2$ and $\tau =\tau _1+\tau _2/2$ from the periodic condition, as shown in Fig. 8b. It has been reported that quadruped animals show this symmetric property in locomotion^41^. The symmetry condition (13) forces the third and fourth rows in (12) to be satisfied and reduces two conditions (this proof is presented in Supplemental Information S5). As a result, the number of independent conditions is reduced to 9 from 11. To find a unique solution, another condition (e.g., total energy) is needed.

### Evaluation of criterion related to spine angular velocity

The solutions we obtained provided a criterion related to spine angular velocity to determine the solution type; the signs of the spine movement angular velocities ${\dot{\phi }}^-$ and ${\dot{\phi }}^+$ are different for one type of flight but identical for two different types of flight. This criterion suggests that the effect of the ground reaction force is too small to change the direction of spine movement in cheetah galloping (Fig. 4). We evaluated the angular velocity changes using measured animal data as follows. The difference of ${\dot{\phi }}^+$ and ${\dot{\phi }}^-$ is given by

14 $$\begin{aligned} \Delta _{{\dot{\phi }}} = {\dot{\phi }}^+-{\dot{\phi }}^- = \frac{d}{j}\Delta _p, \end{aligned}$$

where $\Delta _p = {\dot{y}}^+ - {\dot{y}}^-$ is the vertical impulse at the foot contact. To investigate the ratio of the angular velocity change to the amplitude of the angular velocity, we define

15 $$\begin{aligned} \varepsilon = \frac{\Delta _{{\dot{\phi }}}}{\omega c_1} = \frac{d\Delta _p}{\omega c_1 j}, \end{aligned}$$

which can be calculated using measured animal data, as shown below. When $\varepsilon >1$, solutions have one type of flight. Conversely, solutions with two different flight types have $\varepsilon < 1$.

### Stability analysis

When we found periodic solutions, we computationally investigated the local stability from the eigenvalues of the linearized Poincaré map around the fixed points on a Poincaré section. We defined the Poincaré section by the state just after the second foot contact. Because our model is energy conservative, the gait is asymptotically stable when all of the eigenvalues, except for one eigenvalue of 1, are inside the unit cycle (these magnitudes are less than 1).

### Measurement of animals

To determine the physical parameters (*M*, *L*, and *J*) of our model, we used whole-body computed tomography (CT) from one fresh cadaver of an adult cheetah, obtained from Himeji Central Park (Hyogo, Japan). A total of 1941 consecutive cross-sectional images were obtained using a Supria scanner (Hitachi Medical Corporation, Tokyo, Japan) at the Yamaguchi University Animal Medical Center. The tube voltage and current were set to 120 kV and 200 mA, respectively. The pixel size of each image was 0.841 mm and the slice interval was 0.625 mm. Observations of the spinal oscillation of cheetahs during galloping indicate that the anti-node is located approximately at the 12th thoracic vertebra (T12). Therefore, we divided the CT into the fore and hind parts at T12. We calculated the physical parameters at each part individually and averaged them. To calculate the mass *M*, COM position *L*, and moment inertia *J* around the COM, we approximated the body as multiple elliptical cylinders and assumed that the density is uniform and 1000 kg/$\hbox {m}^3$.

The length of the leg bar *H* indicates the height of the leg root during the stance phase of galloping, and is different from the actual leg length. To determine *H*, we used kinematics data of cheetahs measured during galloping. We examined four adult male cheetahs (40–50 kg) at Shanghai Wild Animal Park (Shanghai, China), who were raised at the zoo from infancy. The cheetahs were encouraged to run around a 400 m track at the zoo using a lure that traveled ahead of them at a speed of 15–18 m/s. Their motion was measured from the side using six high-speed cameras (EX-F1 cameras, CASIO, Tokyo, Japan) at a sampling rate of 600 Hz. We analyzed eight strides during straight running (five from one cheetah and three from the others). We determined *H* from the average of the heights of the shoulder joint and the greater trochanter of the femur during the stance phase.

All procedures used in the present study were performed according to university Guidelines for the Care and Use of Laboratory Animals and were approved by the Ethics and Welfare Committee of Yamaguchi University.

Our model included an impulsive force at the foot contact and the distance *D* determines the position in the model to receive the force. Note that *D* was not determined by the leg root joints of animals. To determine *D*, we used the vertical ground reaction force data of cheetahs measured during galloping in Hudson et al.^36^, as well as the kinematics data above. Specifically, we first calculated the percentage of the stance phase when half of the net impulse during the stance phase was applied in each fore leg and hind leg. We then calculated the horizontal positions of the toe and leg root at the moment from the measured kinematic data in the fore legs and hind legs individually and averaged them to determine *D*.

We determined the spring constant *K* from $\sqrt{2K/J}=2\pi f$, where *f* was the stride frequency determined from the value estimated in  Hudson et al.^36^.

We used the measured amplitudes of the spine oscillation to evaluate the solutions. Specifically, we compared the first amplitude $c_1$ and second amplitude $c_2$ of stable solutions of the types GE and EG in the cheetah model with the measured amplitudes during the first and second flights of cheetahs.

To estimate the ratio of the angular velocity change $\varepsilon$ in (15), we calculated the impulse $\Delta _p$ from the measured ground reaction force data in Hudson et al.^36^ Specifically, we calculated the vertical impulses of each leg individually and averaged them as ${\bar{p}}$. We determined $\Delta _p=2{\bar{p}}$ because two legs touch the ground in one stance phase (Fig. 1). We determined the spine movement amplitude $c_1$ from the measured amplitude of the first flight and $\omega$ from $\omega = \sqrt{H/g}\sqrt{2K/J}$.

## Supplementary Information

Supplementary Information 1
