# Supplementary material for: Dynamical determinants enabling two different types of flight in cheetah gallop to enhance speed through spine movement
Source: Sci Rep. 2021 May 5;11:9631. doi: 10.1038/s41598-021-88879-0 (PMC8099890; doi:10.1038/s41598-021-88879-0)
Supplement: Supplementary file 1 — Supplementary Information 1 [file 41598_2021_88879_MOESM1_ESM.pdf]

Supplementary Information: Dynamical determinants enabling two different types of flight in cheetah gallop to enhance speed through spine movement

Tomoya Kamimura[1,*], Shinya Aoi[2], Yasuo Higurashi[3], Naomi Wada[3], Kazuo Tsuchiya[2], and Fumitoshi Matsuno[4]

[1]Department of Electrical and Mechanical Engineering, Nagoya Institute of Technology, Nagoya, Japan
[2]Department of Aeronautics and Astronautics, Graduate School of Engineering, Kyoto University, Kyoto, Japan
[3]Laboratory of System Physiology, Joint Faculty of Veterinary Medicine, Yamaguchi University, Yamaguchi, Japan
[4]Department of Mechanical Engineering and Science, Graduate School of Engineering, Kyoto University, Kyoto, Japan

[*]kamimura.tomoya@nitech.ac.jp

S1 Effect of assumptions on galloping dynamics

We ignored the pitching movement of the whole body in our model (Fig. 2) because the center of mass vertical and spine joint movements are more important for determining galloping dynamics, compared with pitching movements. This assumption induced simultaneous foot contact between the fore and hind legs. We investigated this dynamical effect based on a model which incorporates θ as the pitch angle of the whole body, as shown in Fig. S1. In this case, the foot contact does not necessarily occur simultaneously between the fore and hind legs.

The motion of this model is governed by the equations of motion of the vertical position of the COM of the whole body Y, whole body pitch angle θ, and spine joint angle ϕ, which are given by

$$\mathcal{K}(\phi)\begin{bmatrix}\ddot{Y}\\ \ddot{\theta}\\ \ddot{\phi}\end{bmatrix}+\begin{bmatrix}0\\ -4ML^2\dot{\theta}\dot{\phi}\cos\phi\sin\phi\\ 4ML^2\dot{\phi}^2\cos\phi\sin\phi\end{bmatrix}+\begin{bmatrix}2Mg\\ 0\\ 4K\phi\end{bmatrix}=E(\theta,\phi)\begin{bmatrix}F_1\\ F_2\end{bmatrix}, \tag{S1}$$

where

$$\mathcal{K}(\phi)=\begin{bmatrix}2M & 0 & 0\\ 0 & 2J+2ML^2\cos^2\phi & 0\\ 0 & 0 & 2J+2ML^2\sin^2\phi\end{bmatrix},$$

$$E(\theta,\phi)=\begin{bmatrix}1 & 1\\ -D\sin\theta\sin\phi-(D+L)\cos\theta\cos\phi & D\sin\theta\sin\phi+(D+L)\cos\theta\cos\phi\\ D\cos\theta\cos\phi+(D+L)\sin\theta\sin\phi & D\cos\theta\cos\phi-(D+L)\sin\theta\sin\phi\end{bmatrix}.$$

F_1 and F_2 are the vertical reaction forces of the fore and hind legs, respectively ($F_i > 0$ for the stance

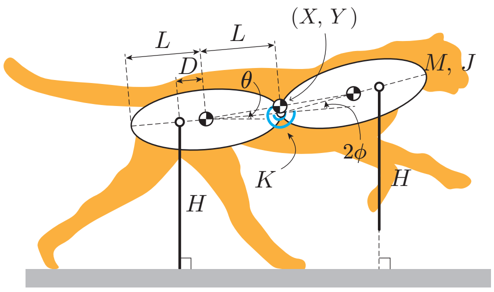

Fig. S1 Model that incorporates the pitch angle θ of the whole body. Foot contacts do not necessarily occur simultaneously between the fore and hind legs.

phase, $F_i = 0$ for the swing phase). The relationship between the states immediately prior to and immediately following the foot contact is given by

$$\mathcal{K}(\phi^-)\begin{bmatrix}\dot{Y}^+ - \dot{Y}^- \\ \dot{\theta}^+ - \dot{\theta}^- \\ \dot{\phi}^+ - \dot{\phi}^-\end{bmatrix} = E(\theta^-, \phi^-)\begin{bmatrix}P_1 \\ P_2\end{bmatrix}, \tag{S2}$$

where P_1 and P_2 are the impulses of the fore and hind legs, respectively ($P_1 > 0$ for the foot contact of the fore leg, $P_2 > 0$ for the foot contact of the hind leg; otherwise $P_i = 0$), and can be determined to satisfy the energy conservation.

We assumed that $|\theta| \ll 1$, $|\phi| \ll 1$, $|\dot{\theta}| \ll 1$, and $|\dot{\phi}| \ll 1$. The linearization of the equations of motion (S1) and relationship between the states immediately prior to and immediately following the foot contact (S2) gives

$$\tilde{\mathcal{K}}\begin{bmatrix}\ddot{y} \\ \ddot{\theta} \\ \ddot{\phi}\end{bmatrix} + \begin{bmatrix}2 \\ 0 \\ 4k\phi\end{bmatrix} = \begin{bmatrix}1 & 1 \\ -(d+l) & d+l \\ -d & -d\end{bmatrix}\begin{bmatrix}f_1 \\ f_2\end{bmatrix}, \tag{S3}$$

$$\tilde{\mathcal{K}}\begin{bmatrix}\dot{y}^+ - \dot{y}^- \\ \dot{\theta}^+ - \dot{\theta}^- \\ \dot{\phi}^+ - \dot{\phi}^-\end{bmatrix} = \begin{bmatrix}1 & 1 \\ -(d+l) & d+l \\ -d & -d\end{bmatrix}\begin{bmatrix}p_1 \\ p_2\end{bmatrix}, \tag{S4}$$

where

$$\tilde{\mathcal{K}} = \begin{bmatrix}2 & 0 & 0 \\ 0 & 2j + 2l^2 & 0 \\ 0 & 0 & 2j\end{bmatrix},$$

$y = Y/H$, $\tau = t/\sqrt{H/g}$, $j = J/(MH^2)$, $k = K/(MgH)$, $d = D/H$, $l = L/H$, $f_i = F_i/Mg$, $p_i = P_i\sqrt{H/g}/(MH)$ $(i = 1, 2)$, and $\dot{*}$ indicates the derivative of variable $*$ with respect to τ. We obtained three equations, each of which included only one of y, θ, or ϕ. While f_1 and f_2 (p_1 and p_2) have different effects (opposite signs) on the equation of θ, they have the same effect on each equation of y and ϕ (same sign). Therefore, the following three cases $f_1 = f_2 = f$ ($p_1 = p_2 = p$), $f_1 = 2f$ and $f_2 = 0$ ($p_1 = 2p$ and $p_2 = 0$), and $f_1 = 0$ and $f_2 = 2f$ ($p_1 = 0$ and $p_2 = 2p$) have the same dynamic effect on y and ϕ.

When $\theta = 0$, the motions of the fore and hind parts of the model are symmetrical, resulting in simultaneous foot contact between the fore and hind legs and $f_1 = f_2 = f$ ($p_1 = p_2 = p$). This effect on y and ϕ is identical to that of individual foot contact between fore and hind legs with $f_1 = 2f$ and $f_2 = 0$ ($p_1 = 2p$ and $p_2 = 0$) and $f_1 = 0$ and $f_2 = 2f$ ($p_1 = 0$ and $p_2 = 2p$). Therefore, even when we ignore the pitching movement ($\theta = 0$), y and ϕ have no significant effect.

S2 Constants of periodic solution

To obtain the periodic solution, we have to determine a_i, b_i, c_i, ψ_i, and τ_i ($i = 1, 2$). We obtained $a_1, a_2, b_1, b_2, c_2, \psi_2, \tau_1$, and τ_2 from the foot-contact conditions (9) and (10), foot-contact relationship (11), periodic conditions (12), and the symmetry assumption (13), as functions of ψ_1 and c_1 as follows:

$$a_1(\psi_1) = \begin{cases} -\dfrac{\psi_1}{\omega}, & -\pi \leq \psi_1 < 0 \\ \dfrac{\pi - \psi_1}{\omega}, & 0 \leq \psi_1 < \pi \end{cases} \tag{S5a}$$

$$a_2(\psi_1, c_1) = \frac{j - d^2}{j + d^2} a_1(\psi_1) - \frac{2jd}{j + d^2} \omega c_1 \sin\psi_1, \tag{S5b}$$

$$b_1(\psi_1, c_1) = b_2(\psi_1, c_1) = 1 - dc_1 \cos\psi_1, \tag{S5c}$$

$$c_2(\psi_1, c_1) = \sqrt{(c_1 \cos\psi_1)^2 + \left(\frac{2d}{j + d^2} \frac{a_1(\psi_1)}{\omega} + \frac{j - d^2}{j + d^2} c_1 \sin\psi_1 \right)^2}, \tag{S5d}$$

$$\cos\psi_2(\psi_1, c_1) = \frac{c_1 \cos\psi_1}{c_2(\psi_1, c_1)}, \tag{S5e}$$

$$\sin\psi_2(\psi_1, c_1) = -\frac{\dfrac{2d}{j + d^2} \dfrac{a_1(\psi_1)}{\omega} + \dfrac{j - d^2}{j + d^2} c_1 \sin\psi_1}{c_2(\psi_1, c_1)}, \tag{S5f}$$

$$\tau_1(\psi_1) = \begin{cases} -\dfrac{2\psi_1}{\omega}, & -\pi \leq \psi_1 < 0 \\ \dfrac{2\pi - 2\psi_1}{\omega}, & 0 \leq \psi_1 < \pi \end{cases} \tag{S5g}$$

$$\tau_2(\psi_1, c_1) = \begin{cases} -\dfrac{2\psi_2(\psi_1, c_1)}{\omega}, & -\pi \leq \psi_2(\psi_1, c_1) < 0 \\ \dfrac{2\pi - 2\psi_2(\psi_1, c_1)}{\omega}, & 0 \leq \psi_2(\psi_1, c_1) < \pi \end{cases}. \tag{S5h}$$

ψ_1 and c_1 satisfy $\Gamma(\psi_1, c_1) = 0$. These functions have physical parameters j, d, and k (this appears as $\omega = \sqrt{2k/j}$).

S3 Parameter dependence of solutions

Here, we show how the types of solutions depend on the impulse position d and the moment of inertia j.

S3.1 When $d \leq -\sqrt{j}$

When the first flight is gathered, $0 \leq \psi_1 < \pi$ is obtained from (1) because $\dot{\phi}(0) < 0$. The substitution of (1) into the fourth row of (11) gives

$$\begin{aligned} \dot{\phi}^+(\tau_1) &= -\frac{2d}{j + d^2} \frac{\psi_1 - \pi}{\omega} + \frac{j - d^2}{j + d^2} \omega c_1 \sin\psi_1 \\ &< 0. \end{aligned} \tag{S6}$$

This indicates that the second flight is also gathered. Therefore, periodic solutions of type GE never exist. From (1) and (S6), $0 \leq \psi_2(\psi_1, c_1) < \pi$ is obtained and (S5) gives

$$\Gamma(\psi_1, c_1) = \frac{\pi - \psi_2(\psi_1, c_1)}{\omega} + \frac{j - d^2}{j + d^2}\frac{\psi_1 - \pi}{\omega} + \frac{2jd}{j + d^2}\omega c_1 \sin\psi_1. \tag{S7}$$

In contrast, (1) gives

$$\dot{y}^+(\tau_1) = -\frac{j - d^2}{j + d^2}\frac{\psi_1 - \pi}{\omega} - \frac{2jd}{j + d^2}\omega c_1 \sin\psi_1. \tag{S8}$$

Because $\Gamma(\psi_1, c_1) = \dfrac{\pi - \psi_2(\psi_1, c_1)}{\omega} - \dot{y}^+(\tau_1)$, $\Gamma(\psi_1, c_1) = 0$ can be satisfied when $\dot{y}^+(\tau_1) > 0$. Therefore, periodic solutions of types G and GG can exist.

When the first flight is extended, $-\pi \leq \psi_1 < 0$ is obtained from (1) because $\dot{\phi}(0) > 0$. The substitution of (1) into the fourth row of (11) gives

$$\dot{\phi}^+(\tau_1) = -\frac{2d}{j + d^2}\frac{\psi_1}{\omega} + \frac{j - d^2}{j + d^2}\omega c_1 \sin\psi_1. \tag{S9}$$

Because the first and second terms of the right-hand side are positive and negative, respectively, the sign of $\dot{\phi}^+(\tau_1)$ and the type of the second flight depend on ψ_1 and c_1. When $\dfrac{\psi_1}{\sin\psi_1} > \dfrac{j - d^2}{2d}\omega^2 c_1$ or when $\dfrac{\psi_1}{\sin\psi_1} = \dfrac{j - d^2}{2d}\omega^2 c_1$ and $-\pi < \psi_1 < -\pi/2$, $\dot{\phi}^+(\tau_1) \leq 0$ is satisfied and the second flight is gathered. However, $0 \leq \psi_2(\psi_1, c_1) < \pi$ is obtained from (1). The substitution of (1) into (S5) gives

$$\begin{aligned}\Gamma(\psi_1, c_1) &= \frac{\pi - \psi_2(\psi_1, c_1)}{\omega} + \frac{j - d^2}{j + d^2}\frac{\psi_1}{\omega} + \frac{2jd}{j + d^2}\omega c_1 \sin\psi_1 \\ &> 0.\end{aligned} \tag{S10}$$

Because $\Gamma(\psi_1, c_1) = 0$ is not satisfied, solutions of type EG never exist. When $\dfrac{\psi_1}{\sin\psi_1} < \dfrac{j - d^2}{2d}\omega^2 c_1$ or when $-\pi/2 < \psi_1 < 0$ and $\psi_2(\psi_1, c_1) = 0$, $\dot{\phi}^+(\tau_1) \geq 0$ is satisfied and the second flight is extended. However, $-\pi \leq \psi_2(\psi_1, c_1) < 0$ is obtained from (1). The substitution of (1) into (S5) gives

$$\begin{aligned}\Gamma(\psi_1, c_1) &= -\frac{\psi_2(\psi_1, c_1)}{\omega} + \frac{j - d^2}{j + d^2}\frac{\psi_1}{\omega} + \frac{2jd}{j + d^2}\omega c_1 \sin\psi_1 \\ &> 0.\end{aligned} \tag{S11}$$

Because $\Gamma(\psi_1, c_1) = 0$ is not satisfied, solutions of types E and EE never exist. When $\dfrac{\psi_1}{\sin\psi_1} = \dfrac{j - d^2}{2d}\omega^2 c_1$ and $\psi_1 = -\pi/2$, $\dot{\phi}^+(\tau_1) = 0$ is satisfied. In this case, $c_2(\psi_1, c_1) = 0$ is obtained from (S5). Therefore, the second flight is neither extended nor gathered (Fig. S2a). However, the substitution of $\dot{\phi}^+(\tau_1) = 0$

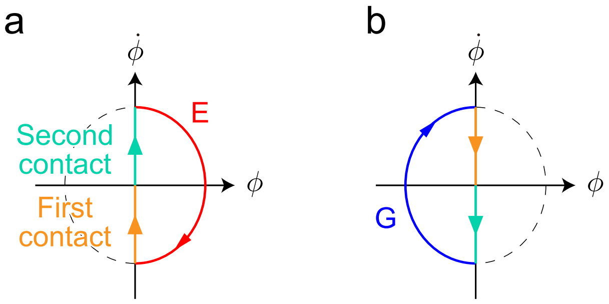

Fig. S2 Periodic solutions whose second flight is neither extended nor gathered. (a) $\psi_1 = -\pi/2$. (b) $\psi_1 = \pi/2$.

and (1) into (S5) gives

$$\Gamma(\psi_1, c_1) = \begin{cases} -\dfrac{\psi_2(\psi_1, c_1)}{\omega} - \dfrac{\pi}{2\omega}\dfrac{j+d^2}{j-d^2} > 0 & -\pi \leq \psi_2(\psi_1, c_1) < 0 \\ \dfrac{\pi - \psi_2(\psi_1, c_1)}{\omega} - \dfrac{\pi}{2\omega}\dfrac{j+d^2}{j-d^2} > 0 & 0 \leq \psi_2(\psi_1, c_1) < \pi \end{cases} \tag{S12}$$

Because $\Gamma(\psi_1, c_1) = 0$ is not satisfied, solutions like Fig. S2a never exist.

Therefore, only solutions of types G and GG can exist when $d \leq -\sqrt{j}$.

S3.2 When $-\sqrt{j} < d < 0$

When the first flight is gathered, $0 \leq \psi_1 < \pi$ is obtained from (1) because $\dot{\hat{\phi}}(0) < 0$. The substitution of (1) into the fourth row of (11) gives

$$\dot{\hat{\phi}}^+(\tau_1) = -\frac{2d}{j+d^2}\frac{\psi_1 - \pi}{\omega} + \frac{j-d^2}{j+d^2}\omega c_1 \sin\psi_1. \tag{S13}$$

Because the first and second terms of the right-hand side are negative and positive, respectively, the sign of $\dot{\hat{\phi}}^+(\tau_1)$ and the type of the second flight depends on ψ_1 and c_1. When $\dfrac{\psi_1 - \pi}{\sin\psi_1} > \dfrac{j-d^2}{2d}\omega^2 c_1$ or when $\dfrac{\psi_1 - \pi}{\sin\psi_1} = \dfrac{j-d^2}{2d}\omega^2 c_1$ and $0 \leq \psi_1 < \pi/2$, $\dot{\hat{\phi}}^+(\tau_1) \geq 0$ is satisfied and the second flight is extended. $-\pi \leq \psi_2(\psi_1, c_1) < 0$ is obtained from (1) because $\dot{\hat{\phi}}^+(\tau_1) \geq 0$. The substitution of (1) into (S5) gives

$$\Gamma(\psi_1, c_1) = -\frac{\psi_2(\psi_1, c_1)}{\omega} + \frac{j-d^2}{j+d^2}\frac{\psi_1 - \pi}{\omega} + \frac{2jd}{j+d^2}\omega c_1 \sin\psi_1. \tag{S14}$$

In contrast, (1) gives

$$\dot{\hat{y}}^+(\tau_1) = -\frac{j-d^2}{j+d^2}\frac{\psi_1 - \pi}{\omega} - \frac{2jd}{j+d^2}\omega c_1 \sin\psi_1. \tag{S15}$$

Because $\Gamma(\psi_1, c_1) = -\dfrac{\psi_2(\psi_1, c_1)}{\omega} - \dot{y}^+(\tau_1)$, $\Gamma(\psi_1, c_1) = 0$ can be satisfied when $\dot{y}^+(\tau_1) > 0$. Therefore, periodic solutions of type GE can exist. When $\dfrac{\psi_1 - \pi}{\sin\psi_1} < \dfrac{j - d^2}{2d}\omega^2 c_1$ or when $\dfrac{\psi_1 - \pi}{\sin\psi_1} = \dfrac{j - d^2}{2d}\omega^2 c_1$ and $\pi/2 < \psi_1 < \pi$, $\dot{\hat{\phi}}^+(\tau_1) \leq 0$ is satisfied and the second flight is gathered. $0 \leq \psi_2(\psi_1, c_1) < \pi$ is obtained from (1) because $\dot{\hat{\phi}}^+(\tau_1) < 0$. The substitution of (1) into (S5) gives

$$\Gamma(\psi_1, c_1) = \frac{\pi - \psi_2(\psi_1, c_1)}{\omega} + \frac{j - d^2}{j + d^2}\frac{\psi_1 - \pi}{\omega} + \frac{2jd}{j + d^2}\omega c_1 \sin\psi_1. \tag{S16}$$

In contrast, (1) gives

$$\dot{y}^+(\tau_1) = -\frac{j - d^2}{j + d^2}\frac{\psi_1 - \pi}{\omega} - \frac{2jd}{j + d^2}\omega c_1 \sin\psi_1. \tag{S17}$$

Because $\Gamma(\psi_1, c_1) = \dfrac{\pi - \psi_2(\psi_1, c_1)}{\omega} - \dot{y}^+(\tau_1)$, $\Gamma(\psi_1, c_1) = 0$ can be satisfied when $\dot{y}^+(\tau_1) > 0$. Therefore, periodic solutions of types G and GG can exist. When $\dfrac{\psi_1 - \pi}{\sin\psi_1} = \dfrac{j - d^2}{2d}\omega^2 c_1$ and $\psi_1 = \pi/2$, $\dot{\hat{\phi}}^+(\tau_1) = 0$ is satisfied. In this case, $c_2(\psi_1, c_1) = 0$ is obtained from (S5). Therefore, the second flight is neither extended nor gathered (Fig. S2b). However, the substitution of $\dot{\hat{\phi}}^+(\tau_1) = 0$, $c_2(\psi_1, c_1) = 0$, and (1) into (S5) gives

$$\Gamma(\psi_1, c_1) = \begin{cases} -\dfrac{\psi_2(\psi_1, c_1)}{\omega} - \dfrac{\pi}{2\omega}\dfrac{j + d^2}{j - d^2} & -\pi \leq \psi_2(\psi_1, c_1) < 0 \\ \dfrac{\pi - \psi_2(\psi_1, c_1)}{\omega} - \dfrac{\pi}{2\omega}\dfrac{j + d^2}{j - d^2} & 0 \leq \psi_2(\psi_1, c_1) < \pi \end{cases} \tag{S18}$$

$\Gamma(\psi_1, c_1) = 0$ is satisfied only when $-\sqrt{j/3} \leq d < 0$. Therefore, periodic solutions like Fig. 2b exist only when $-\sqrt{j/3} \leq d < 0$.

When the first flight is extended, $-\pi \leq \psi_1 < 0$ is obtained from (1) because $\dot{\hat{\phi}}(0) > 0$. The substitution of (1) into the fourth row of (11) gives

$$\begin{aligned} \dot{\hat{\phi}}^+(\tau_1) &= -\frac{2d}{j + d^2}\frac{\psi_1}{\omega} + \frac{j - d^2}{j + d^2}\omega c_1 \sin\psi_1 \\ &> 0. \end{aligned} \tag{S19}$$

This indicates that the second flight is gathered. Therefore, periodic solutions of types E and EE never exist. From (1) and (S19), $0 \leq \psi_2(\psi_1, c_1) < \pi$ is obtained. The substitution of (1) into (S5) gives

$$\Gamma(\psi_1, c_1) = \frac{\pi - \psi_2(\psi_1, c_1)}{\omega} + \frac{j - d^2}{j + d^2}\frac{\psi_1}{\omega} + \frac{2jd}{j + d^2}\omega c_1 \sin\psi_1. \tag{S20}$$

In contrast, (1) gives

$$\dot{y}^+(\tau_1) = -\frac{j - d^2}{j + d^2}\frac{\psi_1}{\omega} - \frac{2jd}{j + d^2}\omega c_1 \sin\psi_1. \tag{S21}$$

Because $\Gamma(\psi_1, c_1) = \dfrac{\pi - \psi_2(\psi_1, c_1)}{\omega} - \dot{y}^+(\tau_1)$, $\Gamma(\psi_1, c_1) = 0$ can be satisfied when $\dot{y}^+(\tau_1) > 0$. From $\dot{y}^+(\tau_1) > 0$, we obtain

$$-\frac{\psi_1}{\omega} > \frac{2jd}{j - d^2}\omega c_1 \sin\psi_1 \tag{S22}$$

From the substitution of (S22) into (S5), we obtain

$$\begin{aligned} c_2(\psi_1, c_1) &> \sqrt{(c_1 \cos\psi_1)^2 + \left(\frac{j + d^2}{j - d^2}\omega c_1 \sin\psi_1\right)^2} \\ &> c_1. \end{aligned} \tag{S23}$$

Because we assumed $c_1 > c_2$, solutions of type EG never exist.

Therefore, only the solutions of types G, GG, and GE, and solutions like Fig. S2b exist when $d < -\sqrt{j}$.

S3.3 When $d = 0$

From (1), $a_2(\psi_1, c_1) = a_1(\psi_1)$, $c_2(\psi_1, c_1) = c_1$, and $\psi_2(\psi_1, c_1) = \psi_1$ are obtained. When the first flight is extended, $-\pi \leq \psi_1 < 0$ is obtained from (1) because $\dot{\hat{\phi}}(0) > 0$. The substitution of (1) into the fourth row of (11) gives

$$\dot{\hat{\phi}}^+(\tau_1) = -\omega c_1 \sin\psi_1 > 0. \tag{S24}$$

This indicates that the second flight is gathered. Therefore, solutions of types E and EE never exist. The substitution of (1) into (S5) gives

$$\Gamma(\psi_1, c_1) = \frac{\pi + 2\psi_1}{\omega}. \tag{S25}$$

$\Gamma(\psi_1, c_1) = 0$ is satisfied when $\psi_1 = -\pi/2$. Therefore, solutions of type EG exist.

When the first flight is gathered, $0 \leq \psi_1 < \pi$ is obtained from (1) because $\dot{\hat{\phi}}(0) < 0$. The substitution of (1) into the fourth row of (11) gives

$$\dot{\hat{\phi}}^+(\tau_1) = -\omega c_1 \sin\psi_1 < 0. \tag{S26}$$

This indicates that the second flight is extended. Therefore, solutions of types G and GG never exist. The substitution of (1) into (S5) gives

$$\Gamma(\psi_1, c_1) = \frac{2\psi_1 - \pi}{\omega}. \tag{S27}$$

$\Gamma(\psi_1, c_1) = 0$ is satisfied when $\psi_1 = \pi/2$. Therefore, solutions of type GE exist.

Therefore, only solutions of types GE or EG exist (they are identical because $c_1 = c_2$).

S3.4 When $0 < d < \sqrt{j}$

Only solutions of types E, EE and EG can exist, in the same way as S3.2.

S3.5 When $\sqrt{j} \leq d$

Only solutions of types E and EE can exist, in the same way as S3.1.

S4 Foot contact dynamics

Here, we derive the relationship (8) between the states immediately prior to and immediately following foot contact in the model. We assumed elastic collision for foot contact, which involves no position change and conserves energy. We define Δ_P as the impulse at foot contact from the ground in the vertical direction. Δ_{P_ϕ} is the change in the angular momentum caused by the impulse. The relationship of the translational and angular momentum between immediately prior to and following the foot contact gives

$$\Delta_P = M(\dot{Y}^+ - \dot{Y}^-) \tag{S28a}$$

$$\Delta_{P_\phi} = J(\dot{\phi}^+ - \dot{\phi}^-) = \Delta_P D \cos\phi \tag{S28b}$$

From energy conservation, we obtain

$$\frac{M}{2}(\dot{Y}^+)^2 + \frac{J}{2}(\dot{\phi}^+)^2 = \frac{M}{2}(\dot{Y}^-)^2 + \frac{J}{2}(\dot{\phi}^-)^2 \tag{S29}$$

From (S28) and (S29), we obtain (8).

S5 Role of symmetry condition in solution

Here, we show the mechanism by which the symmetry condition (13) forces the third and fourth rows in (12) to be satisfied. The substitution of (13) into the first row of (11) gives

$$\hat{y}^-(\tau_1) = \hat{y}(0). \tag{S30}$$

By substituting (1) into (S30), we obtain

$$\dot{\hat{y}}^-(\tau_1) = -\dot{\hat{y}}(0). \tag{S31}$$

By substituting the first and second rows of (12) into (10), we obtain

$$\hat{y}(0) - d\hat{\phi}(0) - 1 = 0. \tag{S32}$$

In contrast, by substituting (S30) into (9), we obtain

$$\hat{y}(0) - d\hat{\phi}^{-}(\tau_1) - 1 = 0. \tag{S33}$$

(S32) and (S33) give

$$\hat{\phi}^{-}(\tau_1) = \hat{\phi}(0). \tag{S34}$$

Because we assumed $\tau_1 < 2\pi/\omega$, the substitution of (1) into (S34) gives

$$\dot{\hat{\phi}}^{-}(\tau_1) = -\dot{\hat{\phi}}(0). \tag{S35}$$

Therefore, from (S30), (S31), (S34), and (S35), the relationship between the states at the beginning of the first flight phase ($\tau = 0$) and immediately prior to the first foot contact ($\tau = \tau_1$) is given by

$$\hat{q}^{-}(\tau_1) = F\hat{q}(0), \tag{S36}$$

where

$$F = \begin{bmatrix} 1 & 0 & 0 & 0 \\ 0 & 1 & 0 & 0 \\ 0 & 0 & -1 & 0 \\ 0 & 0 & 0 & -1 \end{bmatrix}.$$

By substituting (S34) into the second row of (11), we obtain

$$\hat{\phi}^{+}(\tau_1) = \hat{\phi}(0). \tag{S37}$$

The first and second rows of (12), (13), and (S37) give

$$\hat{y}^{-}(\tau_1 + \tau_2) = \hat{y}^{+}(\tau_1), \tag{S38a}$$

$$\hat{\phi}^{-}(\tau_1 + \tau_2) = \hat{\phi}^{+}(\tau_1). \tag{S38b}$$

Because we assume $\tau_2 < 2\pi/\omega$, the substitution of (1) into (S38) gives

$$\dot{\hat{y}}^-(\tau_1 + \tau_2) = -\dot{\hat{y}}^+(\tau_1), \tag{S39a}$$

$$\dot{\hat{\phi}}^-(\tau_1 + \tau_2) = -\dot{\hat{\phi}}^+(\tau_1). \tag{S39b}$$

Therefore, from (S38) and (S39), the relationship between the states immediately following to the first foot contact ($\tau = \tau_1$) and immediately prior to the second foot contact ($\tau = \tau_1 + \tau_2$) is also given by

$$\hat{q}^-(\tau_1 + \tau_2) = F\hat{q}^+(\tau_1). \tag{S40}$$

From (11), (S36), and (S40), we obtain

$$B\hat{q}^-(\tau_1 + \tau_2) = BFBF\hat{q}(0). \tag{S41}$$

Because $BFBF = I$, where I is an identity matrix, $\hat{q}(0) = B\hat{q}^-(\tau_1 + \tau_2)$ is satisfied. This is equal to (12). Therefore, the third and fourth rows of (12) are satisfied when the symmetry condition (13) is given.
